# Interfacial reactions in lithia-based cathodes depending on the binder in the electrode and salt in the electrolyte

**DOI:** 10.1038/s41598-021-04439-6

**Published:** 2022-01-11

**Authors:** Hee Jeong Im, Yong Joon Park

**Affiliations:** Department of Advanced Materials Engineering, Kyonggi University, 154-42, Gwanggyosan-Ro, Yeongtong-Gu, Suwon-Si, Gyeonggi-Do 16227 Republic of Korea

**Keywords:** Energy storage, Materials for energy and catalysis

## Abstract

Lithia (Li_2_O)-based cathodes, utilizing oxygen redox reactions for obtaining capacity, exhibit higher capacity than commercial cathodes. However, they are highly reactive owing to superoxides formed during charging, and they enable more active parasitic (side) reactions at the cathode/electrolyte and cathode/binder interfaces than conventional cathodes. This causes deterioration of the electrochemical performance limiting commercialization. To address these issues, the binder and salt for electrolyte were replaced in this study to reduce the side reaction of the cells containing lithia-based cathodes. The commercially used polyvinylidene fluoride (PVDF) binder and LiPF_6_ salt in the electrolyte easily generate such reactions, and the subsequent reaction between PVDF and LiOH (from decomposition of lithia) causes slurry gelation and agglomeration of particles in the electrode. Moreover, the fluoride ions from PVDF promote side reactions, and LiPF_6_ salt forms POF_3_ and HF, which cause side reactions owing to hydrolysis in organic solvents containing water. However, the polyacrylonitrile (PAN) binder and LiTFSI salt decrease these side reactions owing to their high stability with lithia-based cathode. Further, thickness of the interfacial layer was reduced, resulting in decreased impedance value of cells containing lithia-based cathodes. Consequently, for the same lithia-based cathodes, available capacity and cyclic performance were increased owing to the effects of PAN binder and LiTFSI salt in the electrolyte.

## Introduction

Lithium-ion batteries (LIBs) have attained a status as the most important energy storage device in society. However, widespread usage has led to demands for further enhanced LIBs with higher energy densities, consequently driving the development of high-capacity cathode materials^1–7^. Research conducted in this regard has concluded that the oxygen-related anionic redox reaction may be the key to significantly enhancing the capacity^8–12^. Typical commercial cathodes rely on the cationic redox reaction to realize a certain capacity. The cations within cathodes are transition metals (Co, Ni, Mn, and Fe), and their heavy weight limits the capacity of commercial cathodes. In contrast, some recently reported cathodes utilized the anionic (oxygen) redox reaction based on the oxygen ions in the structure coupled with the cationic redox reaction, which results in a significant increase in the available energy density per unit weight owing to light oxygen ions^13–17^. Moreover, lithia (Li_2_O)-based materials utilizing the pure oxygen redox reaction have been considered as breakthrough cathodes that can accept a capacity higher than any other commercially available cathode^18–20^.

Nevertheless, the commercialization of lithia-based cathodes faces several challenges, including the formation of highly reactive superoxides^21–25^. During the charging process, oxygen ions (O^2−^) in the lithia-based cathodes are oxidized to superoxides (O^x−^, 1 ≤ x < 2) with emitting electrons, and these cause undesirable side reactions with electrolyte or binder, thereby reducing the available capacity as the Li_2_O-derived superoxide is consumed by the side reactions. In addition, parasitic (side) reactions result in the formation of an interfacial layer on the surface of cathodes, which acts as a barrier to the movement of lithium ions and electrons, causing a deterioration in the electrochemical performance of lithia-based cathodes. Moreover, if the Li_2_O-based cathode is charged to an excessive depth, the superoxides are further oxidized, causing the formation of superoxo species (such as O^0.5−^) or the evolution of gaseous O_2_, which leads to structural collapse and seriously degrades the cyclic performance. Fortunately, it is possible to control the excessive charging of lithia-based cathodes by limiting the capacity to the range wherein formation of superoxo species and gaseous oxygen can be prevented. A capacity range, wherein stable cyclic performance can be obtained, is referred to as ‘available capacity’ of lithia-based cathodes. However, side reactions occurring between the charged lithia-based cathodes containing superoxides and electrolyte remains a serious problem yet to be resolved.

Research on suppressing the superoxide-related side reactions has focused on additives^26–28^. Several additives, such as vinylene carbonate (VC), vinylethylene carbonate (VEC), and fluoroethylene carbonate (FEC), can suppress side reactions by forming an organic-based surface coating. However, decreasing the side reactions themselves by replacing certain components of the electrolyte and electrode may be a more efficient strategy. For example, the binder in the electrode and the salt in the electrolyte can considerably affect the side reactions during cycling. In particular, polyvinylidene fluoride (PVDF) and LiPF_6_, which are commercially used as the binder and salt for LIBs, respectively, may be suboptimal components for lithia-based cathodes because they easily activate side reactions. For example, PVDF can trigger slurry gelation by reacting with LiOH, which easily forms on the surface of the lithia-based cathodes^29–31^. In organic solutions containing water, LiPF_6_ hydrolyses into POF_3_ and HF, both of which degrade vulnerable lithia-based cathodes^32,33^.

Therefore, this study compared the electrochemical performance of lithia-based cathodes using two types of binders and two types of salts. As alternatives to PVDF and LiPF_6_, polyacrylonitrile (PAN) and lithium bis(trifluoromethanesulfonyl)imide (LiTFSI) were selected, respectively, because they are less reactive as a binder^34,35^ and salt^36–38^ in LIBs. As a lithia-based cathode, Li_2_O/Li_2_RuO_3_ nanocomposites were used owing to their high capacity and good cyclic performance^23,26^. The optimal combination of binder and salt was determined by comparing the available capacity and cycle life of the Li_2_O/Li_2_RuO_3_ nanocomposites with different binders and salts with the same solvent (ethylene carbonate/dimethyl carbonate, EC/DMC, 1:1 vol%). Furthermore, their effects on the interfacial layer formed on the cathode/electrolyte interface were analysed using transmission electron microscopy (TEM) and X-ray photoelectron spectroscopy (XPS), because this layer critically affects the electrochemical performance of the lithia-based cathodes.

## Electrochemical performance

We prepared the electrodes containing Li_2_O/Li_2_RuO_3_ nanocomposites using two binders (PVDF and PAN), and compared scanning electron microscopy (SEM) images of their surface morphology. For convenience, the electrodes with PVDF and PAN binders are hereafter referred to as the ‘PVDF electrode’ and ‘PAN electrode’, respectively. As shown in Fig. S1, the shape of the electrodes significantly differed depending on the binder. In the PVDF electrode, the Li_2_O/Li_2_RuO_3_ powders appeared to agglomerate into large particles (Fig. S1a). This agglomeration could be attributed to the PVDF binder, which caused the gelation of slurry. The PVDF reacts with LiOH on the cathode surface and forms unsaturated C=C bonds, which polymerize via crosslinking and cause the gelation of the cathode slurry^29–31^. LiOH is formed in significant quantities by decomposition of lithia, thus the slurry gelation by PVDF may be more critical for lithia-based cathodes than other commercial cathodes. In contrast, the PAN electrode consisted of much smaller particles (Fig. S1b), indicating that the PAN binder did not cause the Li_2_O/Li_2_RuO_3_ powders to agglomerate. Large agglomerated particles may prevent the smooth intercalation/deintercalation of the lithium ions from the inside part of electrode. Consequently, using PAN as the binder is expected to be more advantageous in terms of the available capacity than using PVDF.

To compare the electrochemical properties of the Li_2_O/Li_2_RuO_3_ electrodes with different binders, their discharge capacity and cyclic performance were measured. To observe the effect of the electrolyte salts, two types of electrolytes containing LiPF_6_ or LiTFSI salts were also employed, which are hereafter referred to as the ‘LiPF_6_ electrolyte’ and ‘LiTFSI electrolyte’, respectively. Further, the capacity of the cells was limited to 250, 300, and 350 mAh g^−1^ to determine the available capacity which could provide stable cyclic performance without causing the evolution of gaseous oxygen or the formation of superoxo species. The capacity of the Li_2_O/Li_2_RuO_3_ electrodes was calculated based on the weight including both the Li_2_RuO_3_ catalyst and the lithia (active material), although Li_2_RuO_3_ does not contribute to the capacity^23^. Figure S2a–d shows the 1st, 50th, and 100th charge–discharge profiles of the cells at a current density of 100 mA g^−1^ with a limiting capacity of 250 mAh g^−1^. The cells containing both types of electrodes exhibited stable voltage profiles during 100 cycles without a loss of capacity. Furthermore, as shown in Fig. S2e, all cells retained their capacity for 100 cycles.

**Figure 1:**
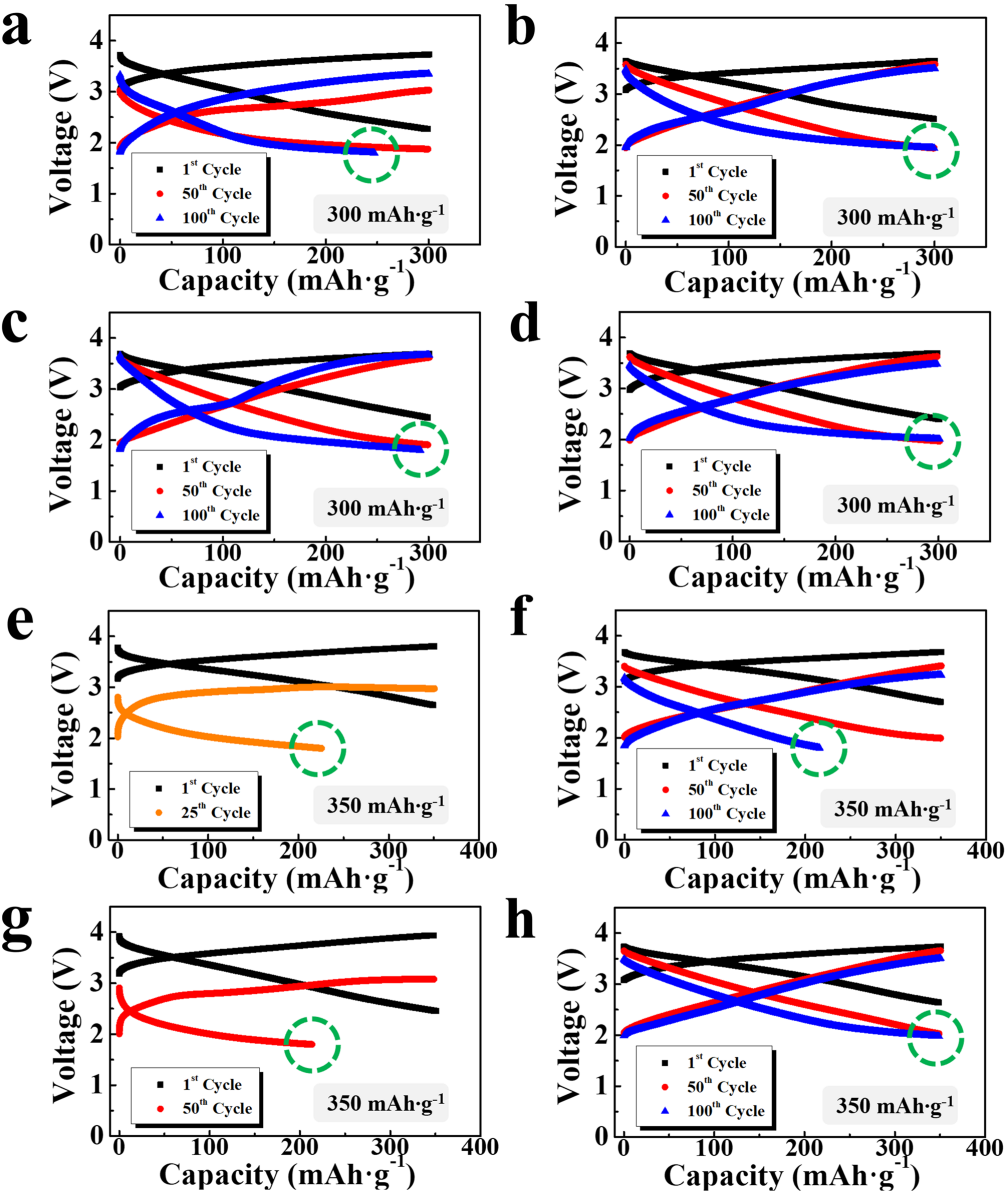
Charge–discharge profiles of the electrodes measured with a limiting capacity of 300 mAh g^−1^ (**a**,**b**) PVDF electrodes cycled using the (**a**) LiPF_6_ and (**b**) LiTFSI electrolytes; (**c**,**d**) PAN electrodes cycled using the (**c**) LiPF_6_ and (**d**) LiTFSI electrolytes; profiles measured with a limiting capacity of 350 mAh g^−1^ (**e**,**f**) PVDF electrodes cycled using the (**e**) LiPF_6_ and (**f**) LiTFSI electrolytes; (**g**,**h**) PAN electrodes cycled using the (**g**) LiPF_6_ and (**h**) LiTFSI electrolytes. Capacity losses are circled in green.

However, when the limiting capacity was increased, a clear difference appeared between the voltage profiles of the cells depending on the binder and salt used in the cells. Figure 1a–d exhibits the 1st, 50th, and 100th charge–discharge profiles of the cells containing PVDF and PAN electrodes with a limiting capacity of 300 mAh g^−1^. With the LiPF_6_ electrolyte, the cells containing the PVDF electrode showed a distinct capacity loss at the 100th cycle (circled in green in Fig. 1a), indicating that the capacity of 300 mAh g^−1^ exceeded the range that can maintain a stable redox reaction during cycling. In contrast, as shown in Fig. 1b, with the LiTFSI electrolyte, the cell containing the PVDF electrode cycled with the same limiting capacity (300 mAh g^−1^) showed a stable voltage profile at the 100th cycle, with no capacity loss. This finding implies that the available capacity can be increased simply by using a suitable salt (LiTFSI) in the electrolyte, without changing the electrode. Meanwhile, with the LiPF_6_ electrolyte, the cell with the PAN electrode exhibited a somewhat more stable voltage profile than that with the PVDF electrode (Fig. 1c), as the 100th discharge profile almost remained at the designated capacity. However, a small amount of capacity loss implies that cells with the PAN electrode and the LiPF_6_ electrolyte also cannot realize stable cyclic performance with the limiting capacity of 300 mAh g^−1^. In contrast, when using the LiTFSI electrolyte, the cell with the PAN electrode showed a stable voltage profile at the 100th cycle, as shown in Fig. 1d.

When the limiting capacity was increased to 350 mAh g^−1^, more pronounced differences appeared, as shown in the 1st, 50th, and 100th charge–discharge profiles of the cells in Fig. 1e–h. When the LiPF_6_ electrolyte was used, the PVDF and PAN electrodes did not retain the designated capacity, even at their respective 50th voltage profiles, clearly indicating that this limiting capacity entered the overcharging region (Fig. 1e,g). In contrast, with LiTFSI, both electrodes showed stable voltage profiles at the 50th cycle, confirming that the LiTFSI electrolyte improved the available capacity of the lithia-based electrodes. However, the PVDF electrode did not maintain a stable capacity for 100 cycles, exhibiting a severe capacity loss (Fig. 1f). In contrast, the PAN electrode and the LiTFSI electrolyte maintained a stable voltage profile at the 100th cycle (Fig. 1h), thus confirming that the PAN electrode had a higher available capacity than the PVDF electrode.

**Figure 2:**
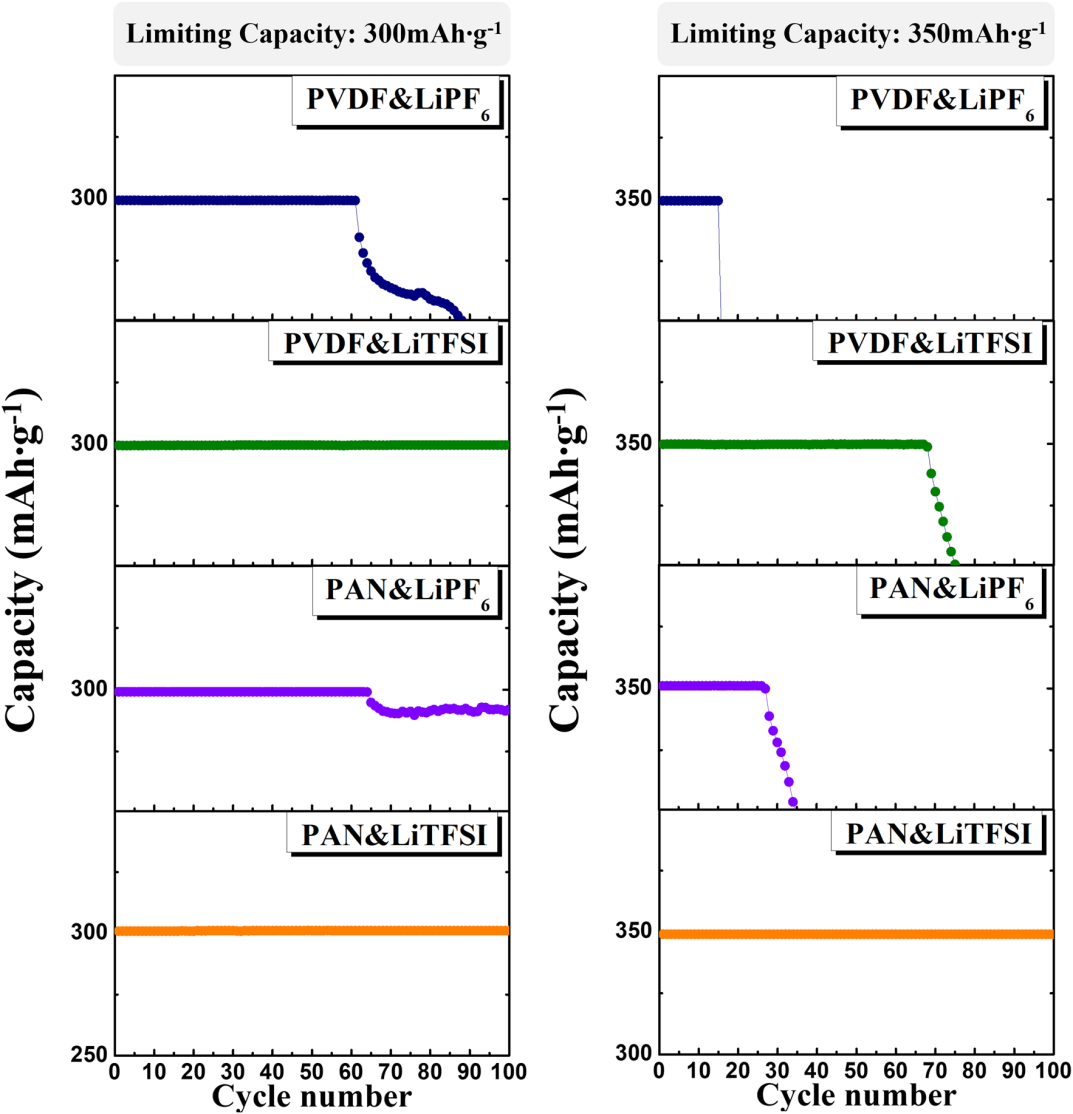
Cyclic performance of the electrodes measured using the LiPF_6_ or LiTFSI electrolyte with limiting capacities of 300 mAh g^−1^ (left) and 350 mAh g^−1^ (right).

Figure 2 shows the cyclic performance of the cells containing PVDF and PAN electrodes measured with the two types of electrolytes (LiPF_6_ and LiTFSI). With the limiting capacity of 300 mAh g^−1^ (left side), the cells with LiPF_6_ start to lose their capacity after 62–67 cycles, but with LiTFSI, they retained the designated capacity for 100 cycles. However, when the limiting capacity was increased to 350 mAh g^−1^ (right side), only the cell containing the PAN electrode and the LiTFSI electrolyte remained stable for 100 cycles. The capacity losses of the other cells during cycling confirmed that 350 mAh g^−1^ is in the overcharged range for the cells.

The lower available capacity of the PVDF electrode than that of the PAN electrode may be associated with the agglomerated cathode particles caused by slurry gelation through the dehydrofluorination of the PVDF binder^29–31^. The lithium ions located inside the agglomerated cathode may not have been able to escape smoothly during the charging process. Eventually, although the interior of the cathode was not sufficiently charged, the lithium ions on the surface of the cathode disproportionately deintercalated and more quickly reached an excessive charge depth, which could form superoxo species. Thus, even at a lower limiting capacity, capacity could fade owing to the excessive charging of the surface region of the cathode. In contrast, the lithium ions in the PAN electrode, which maintained smaller particles, could easily move from the electrode interior to the surface during the charging process, which may have enabled a somewhat higher available capacity as the surface region could more slowly reach this excessive charge depth. Moreover, the possibility that fluoride ions from the PVDF promoted side reactions and decreased the available capacity cannot be excluded.

The higher available capacity of the cell with the LiTFSI electrolyte compared with that using the LiPF_6_ electrolyte can be attributed to the higher stability of the LiTFSI salt. Although the LiPF_6_ salt forms POF_3_ and HF, which, in turn, cause side reactions with cathodes via hydrolysis in the organic solvent containing water, LiTFSI exhibits a higher tolerance to water and does not easily decompose in the electrolyte. In particular, lithia-based cathodes are more vulnerable than commercial cathodes; thus, the effect of side reactions accelerated by the decomposition of salt may be more critical.

## Characterization of interfacial reaction

To observe the interfacial reaction that occurs depending on the binder in more detail, the surface of the PVDF and PAN electrodes was examined before and after cycling using SEM, TEM and XPS. Figure S3 presents the TEM images of the pristine electrodes prior to the electrochemical test (i.e. without contact with the electrolyte). Both PVDF and PAN electrodes had clean surfaces, and no reaction layers were observed. Figure S4 shows the XPS spectra of the electrodes before such testing. The C 1 s spectrum of the PVDF electrode in Fig. S4a revealed the presence of C–C bonds (~ 284.5 eV) owing to carbon^39,40^, C–F_2_ (~ 290.5 eV) and C–H_2_ (~ 285.7 eV) bonds attributed to the PVDF binder^26^, and C–O–C (~ 287.1 eV) bonds related to the residual carbon impurities^41^. Further, the F 1 s spectrum of the PVDF electrode exhibits a large peak related to LiF (~ 685.0 eV), corresponding to the reaction between lithia and PVDF binder, as well as a C–F_2_ peak (~ 687.9 eV) owing to the binder^26^ (Fig. S4b). Notably, the XPS spectrum of the PAN electrode was significantly different from that of the PVDF electrode. As shown in Fig. S4c, although the C 1 s spectrum also demonstrated C–C (~ 284.5 eV), C–H_2_ (~ 285.7 eV), and C–O–C (~ 287.1 eV) bonds, CH–CN (~ 285.2 eV) and C≡N (~ 286.6 eV) bonds were also detected because of the PAN binder. In addition, the Li_2_CO_3_ (~ 289.8 eV) and CO_2_ (~ 288.6 eV) peaks may have been associated with the reaction between the PAN binder and residual impurities on the surface. The F 1 s spectrum of the PAN electrode (Fig. S4d) was even more impressive; specifically, the LiF and C–F_2_ peaks in the F 1 s spectrum of the PVDF electrode did not appear, implying that the side reactions related to the fluoride ions were perfectly suppressed.

Figure S5a–d presents SEM and TEM images of the PVDF electrode after cycling using the LiPF_6_ electrolyte. The cycling capacity was limited to 300 mAh g^−1^, while the current density was 100 mA g^−1^. In the SEM images, most of the cathode particles still appear agglomerated after 1 and 100 cycles (Fig. S5a,b). However, the large-sized particles appeared to have been reduced after cycling, which may imply that the agglomerated particles could disperse to a certain extent during cycling. Further, the TEM images clearly reveal that the surfaces change during cycling. As shown in Fig. S5c–d, the cycled surface of the PVDF electrodes was covered with a thick interfacial layer, which was due to parasitic (side) reactions between the lithia-based cathode and the LiPF_6_ electrolyte. The XPS spectra of the cycled PVDF electrodes also showed new peaks related to parasitic reactions during cycling. The C 1 s spectrum (Fig. S6a–b) revealed the presence of Li_2_CO_3_ (~ 289.8 eV) and –CO_2_ (~ 288.6 eV) bonds, which are associated with the decomposition of carbonate solvents, whereas the F 1 s spectrum (Fig. S6c–d) showed a Li_x_PO_y_F_z_ peak (~ 686.9 eV), attributed to the dissociation of LiPF_6_ salt. Overall, it seemed that the parasitic reactions on the surface of the lithia-based cathode were more active than those occurring in commercialized cathode materials, which could be attributed to the high reactivity of charged lithia-based cathodes containing Li_2_O_2_ or superoxo species.

**Figure 3:**
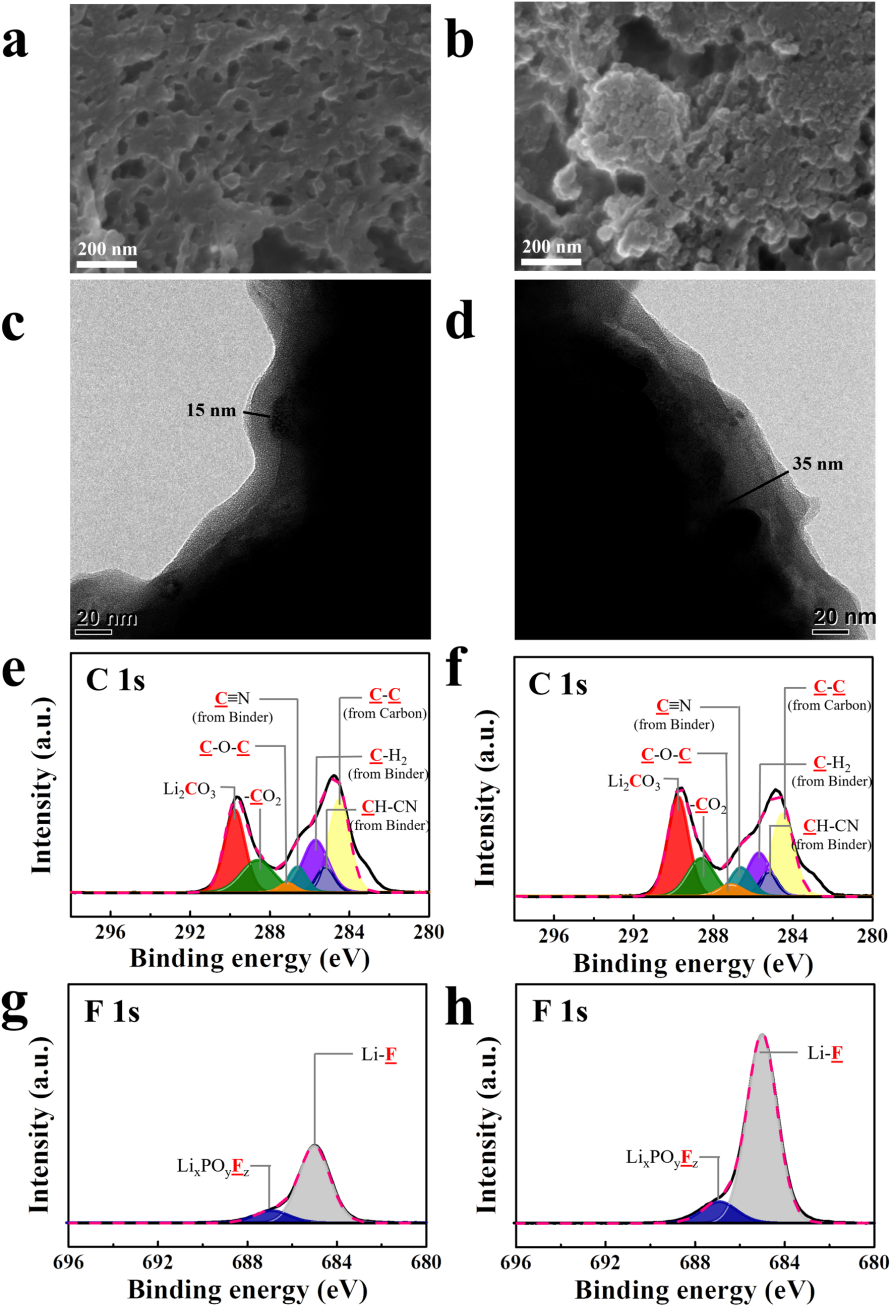
Characterization of cells with PAN electrodes cycled using the LiPF_6_ electrolyte with a limiting capacity of 300 mAh g^−1^. SEM images after the (**a**) 1st and (**b**) 100th cycle; TEM images after the (**c**) 1st and (**d**) 100th cycle; XPS spectra: C 1 s spectra after the (**e**) 1st and (**f**) 100th cycles; F 1 s spectra after the (**g**) 1st and 100th cycles.

Figure 3a–d shows SEM and TEM images of the PAN electrode after cycling using the LiPF_6_ electrolyte. The SEM images of the PAN electrode after cycling reveal that the surface morphology did not significantly change with respect to the surface before testing (Fig. 3a–b). Notably, according to the TEM images, the interfacial layer formed on the surface of the PAN electrode after cycling was much thinner (Fig. 3c–d) than that on the surface of the PVDF electrode (Fig. S5c–d), although the cathode powder (Li_2_O/Li_2_RuO_3_ nanocomposite) and electrolyte are the same. Thus, PAN as the binder is clearly advantageous in mitigating interfacial parasitic reactions. However, the interfacial layer after the 1st cycle (~ 15 nm) was clearly thicker after the 100th cycle (~ 35 nm). Further, the C 1 s spectrum of the PAN electrode after cycling (Fig. 3e) did not significantly change with respect to that of the electrode before testing (Fig. S4c), except for a small increase in the amount of –CO_2_ bonds. However, in the F 1 s spectrum, LiF (685 eV) and Li_x_PO_y_F_z_ (~ 686.9 eV) peaks appeared after the first cycle, which were due to a parasitic reaction related to the LiPF_6_ salt. In addition, the intensity of these new peaks was higher after 100 cycles, which may have been related to the thicker interfacial layer.

**Figure 4:**
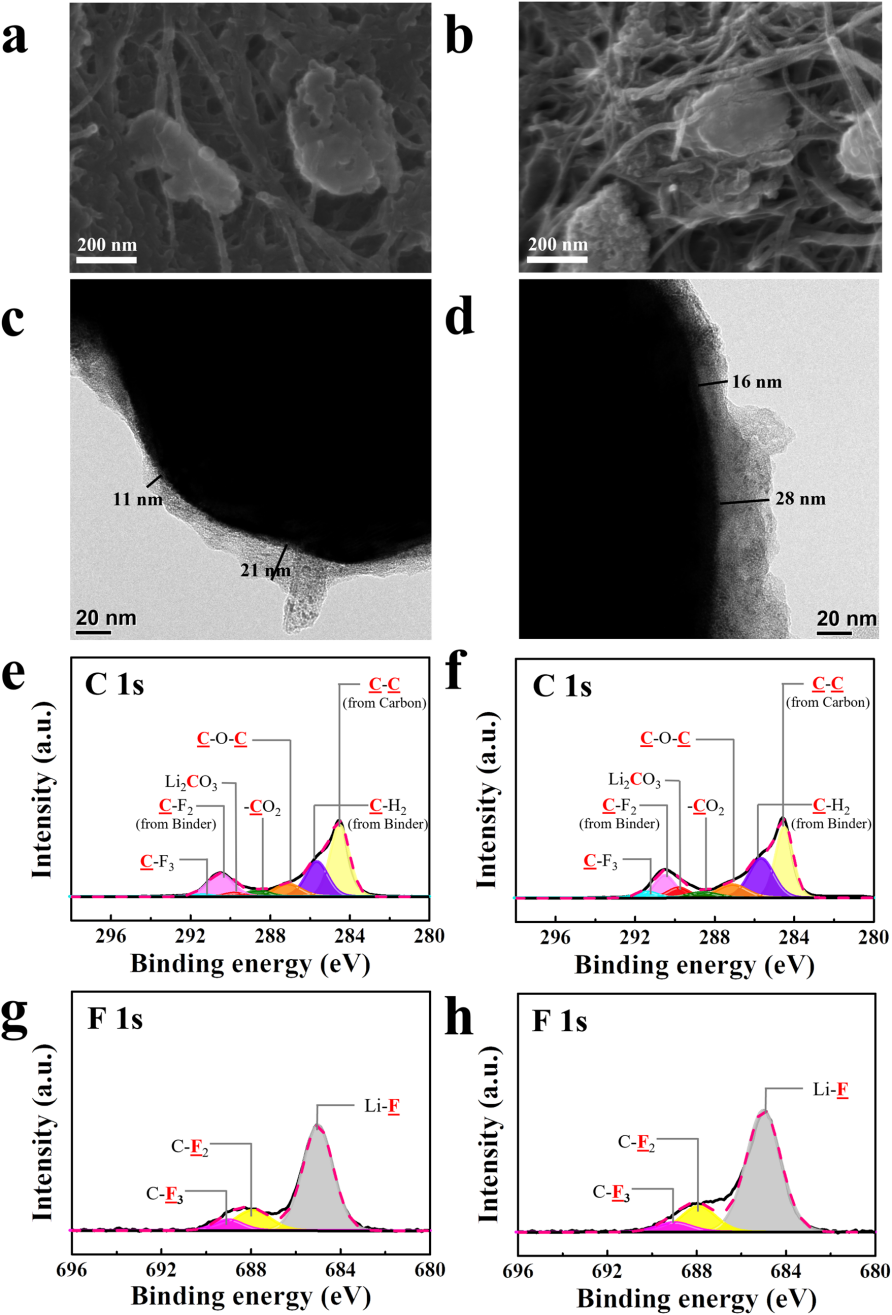
Characterization of cells with PVDF electrodes cycled using the LiTFSI electrolyte with a limiting capacity of 300 mAh g^−1^. SEM images after (**a**) 1st and (**b**) 100th cycle; TEM images after (**c**) 1st and (**d**) 100th cycle; XPS spectra: C 1 s spectra after the (**e**) 1st and 100th cycles; F 1 s spectra after the (**g**) 1st and (**h**) 100th cycles.

These results clearly show that parasitic reactions associated with lithia-based cathodes depend on the electrode binder, which is believed to cause the difference in the electrochemical properties between the PVDF and PAN electrodes, along with the agglomeration effect of the PVDF binder. However, the electrochemical properties of the electrodes were also highly dependent on the salt in the electrolyte, as shown in Figs. 1 and 2. To observe the change in the interfacial reactions according to the salt in the electrolyte, the SEM, TEM, and XPS analyses were performed on the cycled PVDF and PAN electrodes using the LiTFSI electrolyte. Figure 4a–d shows the SEM and TEM images of the PVDF electrode after cycling with this electrolyte. The SEM images demonstrate that this electrode also contained large agglomerated particles (Fig. 4a–b); however, compared with the morphology of the PVDF electrode before testing (Fig. S1a), the particles appeared to be considerably dispersed during cycling. The TEM images indicate that the interfacial layer was much thinner (Fig. 4c–d) than that formed during cycling using the LiPF_6_ electrolyte (Fig. S5c–d), which indicates that the parasitic reactions were somewhat mitigated by using LiTFSI instead of LiPF_6_ as the salt. However, the slight increase in the thickness of the interfacial layer after 100 cycles implies that the parasitic reaction continued to some degree during cycling. Interestingly, the thickness of the interfacial layer on the PVDF electrode using the LiTFSI electrolyte was similar to that on the PAN electrode cycled using the LiPF_6_ electrolyte (Fig. 3c–d). However, the former surface layer appeared more uneven and less stable than the latter.

Figure 4e–h shows the XPS spectra of the PVDF electrode after cycling with the LiTFSI electrolyte. The C 1 s spectrum was approximately similar to that using the LiPF_6_ electrolyte (Fig. S6), except for the intensity of the C–C peak attributed to carbon (conducting agent), which was due to the inhomogeneous distribution of carbon. In contrast, the F 1 s spectra were significantly different. In particular, the Li_x_PO_y_F_z_ peak (~ 686.9 eV) was not detected for the PVDF electrode with the LiTFSI electrolyte. Considering that Li_x_PO_y_F_z_ is, a by-product obtained from the decomposition of LiPF_6_, and HF, deteriorating the cathodes, is generated in this process, its absence exerts a positive effect on the performance of lithia-based cathodes. Furthermore, although the LiF peak appeared, it was less intense than that in the case of the LiPF_6_ electrolyte. This result implies that the LiTFSI electrolyte led to fewer side reactions accompanied by the generation of HF than the LiPF_6_ electrolyte, which could thus mitigate the deterioration of lithia-based cathodes during cycling and enhance their electrochemical performance, as shown in Figs. 1 and 2. The weak C–F_3_ peak (~ 689.0 eV) could be attributed to the residual LiTFSI salt^41^.

**Figure 5:**
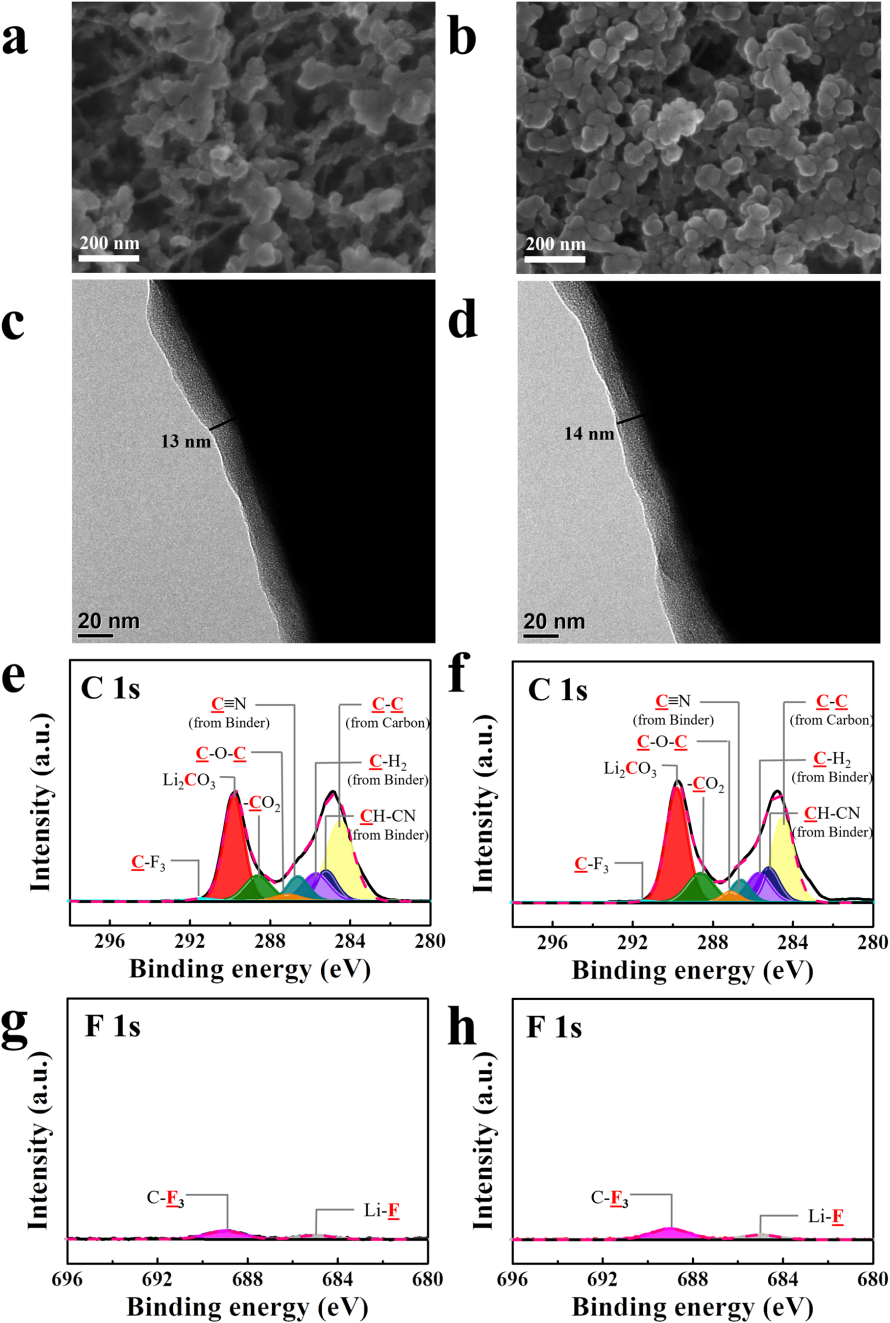
Characterization of cells with PAN electrodes cycled using the LiTFSI electrolyte with a limiting capacity of 300 mAh g^−1^. SEM images after the (**a**) 1st and (**b**) 100th cycles; TEM images after the (**c**) 1st and (**d**) 100th cycles; XPS spectra: C 1 s spectra after the (**e**) 1st and (**f**) 100th cycles; F 1 s spectra after the (**g**) 1st and (**h**) 100th cycles.

Figures 3 and 4 confirm the benefits of PAN as the binder and LiTFSI as the salt for lithia-based cathodes, suggesting that simultaneously using these two components is optimal. Figure 5a–d presents the SEM and TEM images of the PAN electrode after cycling using the LiTFSI electrolyte. The SEM images after cycling (Fig. 5a,b) do not reveal clear differences from the PAN electrode cycled with the LiPF_6_ electrolyte, but in the TEM image (Fig. 5c,d), the interfacial layer appears to be thin and homogeneous. In addition, its thickness barely increased during cycling, which is in contrast with layers that formed under the other conditions, where the thickness of the interfacial layer clearly increased during cycling, as shown in Figs. S5, 3, and 4. However, when the PAN electrode was cycled using the LiTFSI electrolyte, the interfacial layer hardly grew, indicating that parasitic (side) reactions related to the interfacial layer did not actively proceed during cycling. Figure 5e–h shows the XPS spectra of the PAN electrode cycled using the LiTFSI electrolyte, which verify this result. In the C 1 s spectrum, although the peak intensity of the Li_2_CO_3_ and –CO_2_ bonds was somewhat higher than those for the pristine PAN electrode before testing (Fig. S4), the peaks did not change after 100 cycles. Moreover, in the F 1 s spectrum, except for the C–F_3_ peak (~ 689.0 eV) due to residual LiTFSI salt, almost no distinct peak was detected, even after 100 cycles. Although a weak LiF peak was observed, its intensity was negligible, and it did not increase in intensity during cycling. Figure S7 compares the F 1 s spectra of the electrodes after 1 and 100 cycles, indicating that when the PVDF and PAN electrodes were cycled using the LiPF_6_ electrolyte, the LiF and Li_x_PO_y_F_z_ peaks increased during cycling. Further, although the LiF peak was relatively indistinct, when the PVDF electrode was cycled using the LiTFSI electrolyte, the intensity of this peak increased. Only in the case of the PAN electrode cycled using the LiTFSI electrolyte were a few differences detected, which can explain the constant thickness of the interfacial layer during cycling and the good electrochemical performance of cells with this combination, as shown in Figs. 1 and 2.

**Figure 6:**
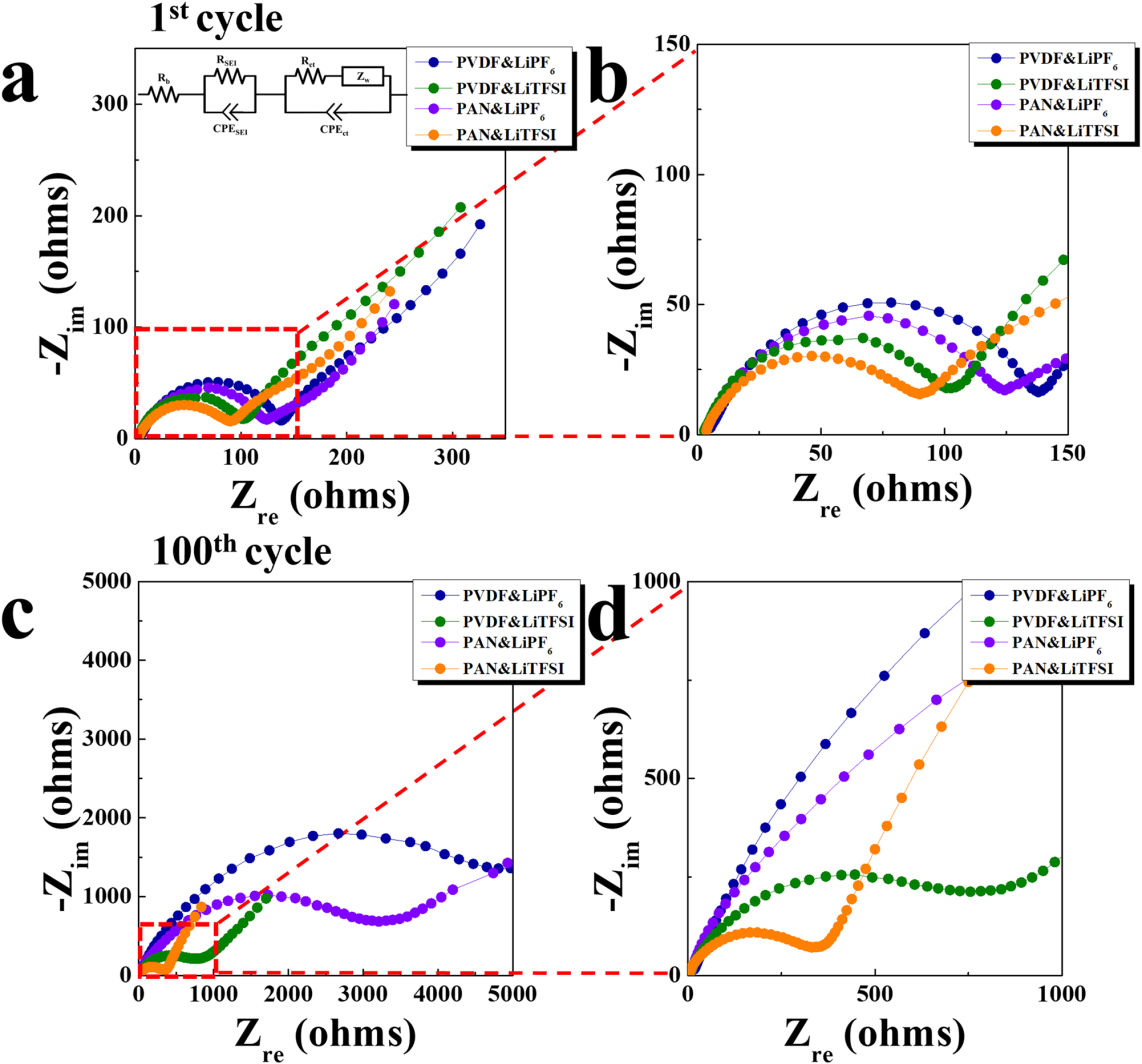
Nyquist plots of the cells containing PVDF or PAN electrodes using LiPF_6_ or LiTFSI electrolyte with a limiting capacity of 300 mAh g^−1^: (**a**) and (**b**) after the 1st cycle and (**c**) and (**d**) after the 100th cycle (inset in (**a**): equivalent circuit for the fitting).

The impedance of the cells containing PVDF and PAN electrodes was also examined to characterize the effect of the electrode binder and the electrolyte salt. Figure 6 shows the Nyquist plots of the four types of cells after 1 and 100 cycles. After one cycle, the semicircle for the cells containing LiPF_6_ electrolyte was slightly larger than that for the cells containing LiTFSI electrolyte, indicating that the LiPF_6_ salt increased the impedance value with respect to the case of the LiTFSI electrolyte. Moreover, when the cells were cycled 100 times, the semicircle for the cells containing LiPF_6_ electrolyte significantly increased. Although the cells containing the LiTFSI electrolyte showed a wider semicircle as well, this increase was smaller. Furthermore, the cells containing the PAN electrode showed smaller semicircles than cells containing the PVDF electrode. For a more detailed analysis, the Nyquist plots were fitted based on the equivalent circuit illustrated in the inset of Fig. 6a, and the resistance values are summarized in Table 1. R_SEI_ is the resistance attributed to the solid electrolyte interphase (SEI) layer, and R_ct_ is the charge transfer resistance. Further, R_b_ and W represent the bulk and Warburg resistances, respectively. The R_SEI_ and R_ct_ values for the cell containing the PVDF electrode and LiPF_6_ electrolyte were 5.5 and 132.7 Ω, respectively. When the LiPF_6_ electrolyte was replaced by the LiTFSI electrolyte, these values for the cell containing the PVDF electrode decreased to 3.1 (R_SEI_) and 102.9 Ω (R_ct_). In addition, the decrease in impedance owing to the LiTFSI electrolyte was more distinct after 100 cycles. The cells containing the PVDF electrode and LiPF_6_ electrolyte yielded high R_SEI_ (30.8 Ω) and R_ct_ (4233.1 Ω) values. However, with the LiTFSI electrolyte, the PVDF electrode cell showed much lower R_SEI_ (7.6 Ω) and R_ct_ (713.9 Ω) values. This again confirms that using the LiTFSI electrolyte hinders the parasitic reactions that generate the interfacial layer, which, in turn, decreases the impedance of the cell with respect to that of the cell using the LiPF_6_ electrolyte. Moreover, the impedance also decreased when the PAN electrode was used instead of the PVDF electrode. The R_SEI_ and R_ct_ values for the cell using the PAN electrode and LiTFSI electrolyte after 100 cycles were only 4.4 and 331.7 Ω, respectively, which indicates the higher stability of the PAN binder than that of the PVDF binder. This finding demonstrates that the superior electrochemical performance of the cell containing the PAN electrode and LiTFSI electrolyte is due to the synergic effects of the stable PAN binder and LiTFSI salt when they are applied in lithia-based cathodes.

**Table 1:** Impedance values derived from the Nyquist plots for cells containing PVDF or PAN electrode after the 1st and 100th cycle using the LiPF_6_ or LiTFSI electrolyte.

Resistance	After the 1st cycle			After the 100th cycle		
	R_b_ (Ω)	R_SEI_ (Ω)	R_ct_ (Ω)	R_b_ (Ω)	R_SEI_ (Ω)	R_ct_ (Ω)
Cell components						
PVDF and LiPF_6_	5.2	5.5	132.7	17.7	30.8	4233.1
PVDF and LiTFSI	2.7	3.1	102.9	5.7	7.6	713.9
PAN and LiPF_6_	3.4	4.0	122.4	8.5	17.9	2490.8
PAN and LiTFSI	3.5	4.0	90.6	3.8	4.4	331.7

## Summary

To reduce the parasitic reactions of the lithia-based cathode, which has high capacity but very highly reactivity, commercially used PVDF binder and LiPF_6_ salt for electrolyte were replaced by PAN (binder) and LiTFSI (salt). The PVDF electrode showed large agglomerated particles, attributed to the crosslinking polymerization of C=C unsaturated bonds owing to the reaction between PVDF and LiOH. LiOH easily formed on the surface of the lithia-based cathode owing to the decomposition of lithia; thus, this parasitic reaction is more active for lithia-based cathodes than for other commercial cathodes. In contrast, the electrode using the PAN binder was composed of smaller particles, which is advantageous to the electrochemical performance because the lithium ions in the electrode interior can be smoothly intercalated/deintercalated during cycling. After cycling using the LiPF_6_ electrolyte, the PVDF electrode formed a thick interfacial layer, whereas the PAN electrode had a relatively thinner, more homogeneous interfacial layer. Parasitic reactions were thus mitigated by using the PAN binder, which increased the available capacity and decreased the impedance of the PAN electrode with respect to those of the PVDF electrode.

**Figure 7:**
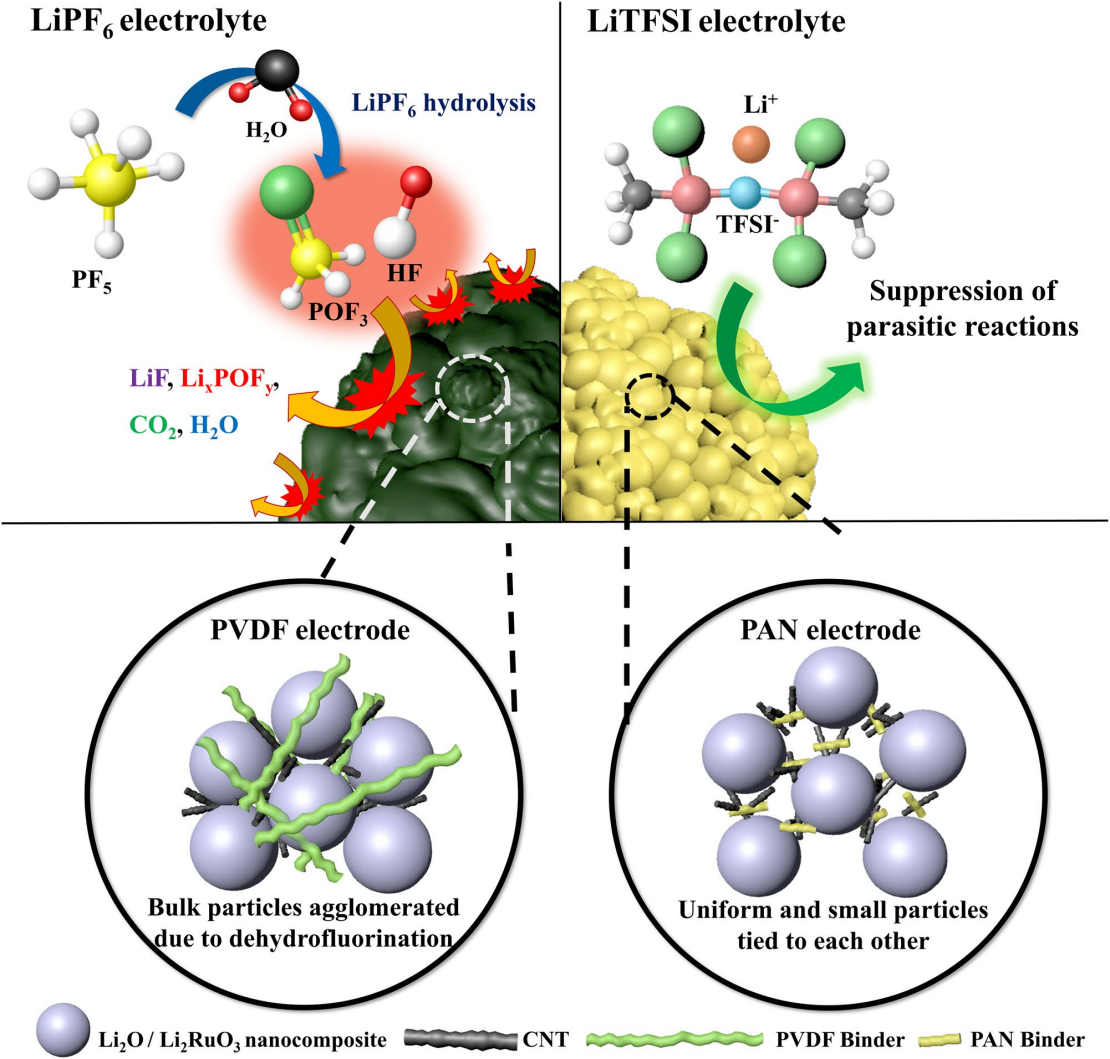
Schematic illustrating the synergic effects of the PAN binder and LiTFSI electrolyte salt on lithia-based cathodes.

Furthermore, replacing the salt in the electrolyte caused a critical change in the properties of the electrode containing the lithia-based cathode. In the XPS analysis, large LiF and Li_x_PO_y_F_z_ peaks were detected in the XPS spectra of the electrodes cycled using the LiPF_6_ electrolyte, which were due to parasitic reactions related to the LiPF_6_ salt. However, the XPS spectra of the electrode cycled using the LiTFSI electrolyte did not exhibit Li_x_PO_y_F_z_ peaks. Because Li_x_PO_y_F_z_ forms from the decomposition of LiPF_6_ and is related to the generation of HF, which can damage the cathode, the absence of Li_x_PO_y_F_z_ indicates that replacing this salt with LiTFSI eliminated the corresponding side reaction. Consequently, the interfacial layer of the electrodes cycled using the LiTFSI electrolyte was thinner than that of the electrodes using the LiPF_6_ electrolyte, and the available capacity was greatly increased by using the LiTFSI electrolyte. Moreover, the PAN electrode cycled using the LiTFSI electrolyte showed a much higher available capacity and more stable cyclic performance owing to the mitigation of parasitic reactions derived from the synergic effect of the stable PAN binder and LiTFSI salt. Figure 7 summarizes the effects of the PAN binder and LiTFSI salt on the reactive lithia-based cathode.

## Methods

We prepared Li_2_O/Li_2_RuO_3_ nanocomposites as a lithia-based cathode according to a previously reported method^23^. We fabricated Li_2_RuO_3_ as a catalyst from RuO_2_ (Aldrich, 99.9%) and Li_2_CO_3_ (Aldrich, 99.9%) in a ratio of 1:1 (mol%) through a sintering process at 950 °C for 24 h in air. Then, we dispersed the synthesized Li_2_RuO_3_ with lithia (Li_2_O) in butanol, and adjusted the Ru content [f_Ru_ = Ru/(Ru + Li)] to 0.09 mol %. Next, we filtered the resulting solution and dried it under vacuum at 80 °C for 24 h. Subsequently, we sealed the dried powder within a milling container with zirconia balls (5 and 10 mm diameters, 1:1 wt%) in an Ar-filled glove box. We ball-milled this powder at 600 rpm for a total of 150 h (milling for 1 h 30 min followed by a 30-min rest, then repeating this 2-h cycle) using a planetary mill (Pulverisette 6, Fritch) to obtain the Li_2_O/Li_2_RuO_3_ nanocomposites.

For the electrochemical tests, we prepared the cathode by mixing the active material (Li_2_O/Li_2_RuO_3_ nanopowder), carbon nanotubes, and binder (60:30:10 wt%) in *N*-methyl-2-pyrrolidone (NMP) solvent. The selected binder was either PVDF or PAN. We assembled the cathode into a 2032 coin cell with a Li-metal anode, an electrolyte, and a Celgard 2400 separator. Further, the solvent of electrolyte was a mixture of EC and DMC (1:1 vol%). The electrolyte contained either 1 M LiPF_6_ or LiTFSI salt dissolved in the EC/DMC solvent. We conducted the cycling tests with a current density of 100 mA∙g^−1^ in the potential range of 1.8–4.35 V using a WonATech voltammetry system. We measured the impedance using an electrochemical workstation (Ametak, VersaSTAT 3) by applying an alternating current (AC) voltage with an amplitude of 5 mV over a frequency range of 0.1 Hz to 100 kHz.

To observe the surface morphology of the cathode before testing and after the 1st and 100th cycles, we performed TEM (JEOL JEM-2100F, Cs corrector). In addition, we conducted SEM (JEOL JSM-7610F PLUS) analysis to characterize the overall form of the Li_2_O/Li_2_RuO_3_ cathode depending on the binder and electrolyte. Further, to confirm the electrochemical reaction of the cells, we analysed the surface of the Li_2_O/Li_2_RuO_3_ cathode using XPS (NEXSA, Thermo Scientific K-Alpha^+^). We rinsed all the samples with DMC to wash away the salt contained in the electrolyte. We fitted the obtained XPS spectra using XPS peak software (Avantage Data System), and calibrated the binding energy scale using the C–C peak (284.5 eV) in the C 1 s spectrum.

## Supplementary Information

Supplementary Information.
