# Supplementary material for: Interfacial reactions in lithia-based cathodes depending on the binder in the electrode and salt in the electrolyte
Source: Sci Rep. 2022 Jan 11;12:527. doi: 10.1038/s41598-021-04439-6 (PMC8752660; doi:10.1038/s41598-021-04439-6)
Supplement: Supplementary file 1 — Supplementary Information. [file 41598_2021_4439_MOESM1_ESM.docx]

**Supplementary Information**

**Interfacial reactions in lithia-based cathodes depending on the binder in the electrode and salt in the electrolyte**

Hee Jeong Im and Yong Joon Park*

Department of Advanced Materials Engineering, Kyonggi University, 154-42, Gwanggyosan-Ro, Yeongtong-Gu, Suwon-Si, Gyeonggi-Do 16227, Republic of Korea

^*^Corresponding author

Ph: +82-31-249-9769; E-mail: [yjpark2006@kyonggi.ac.kr](mailto:yjpark2006@kyonggi.ac.kr)


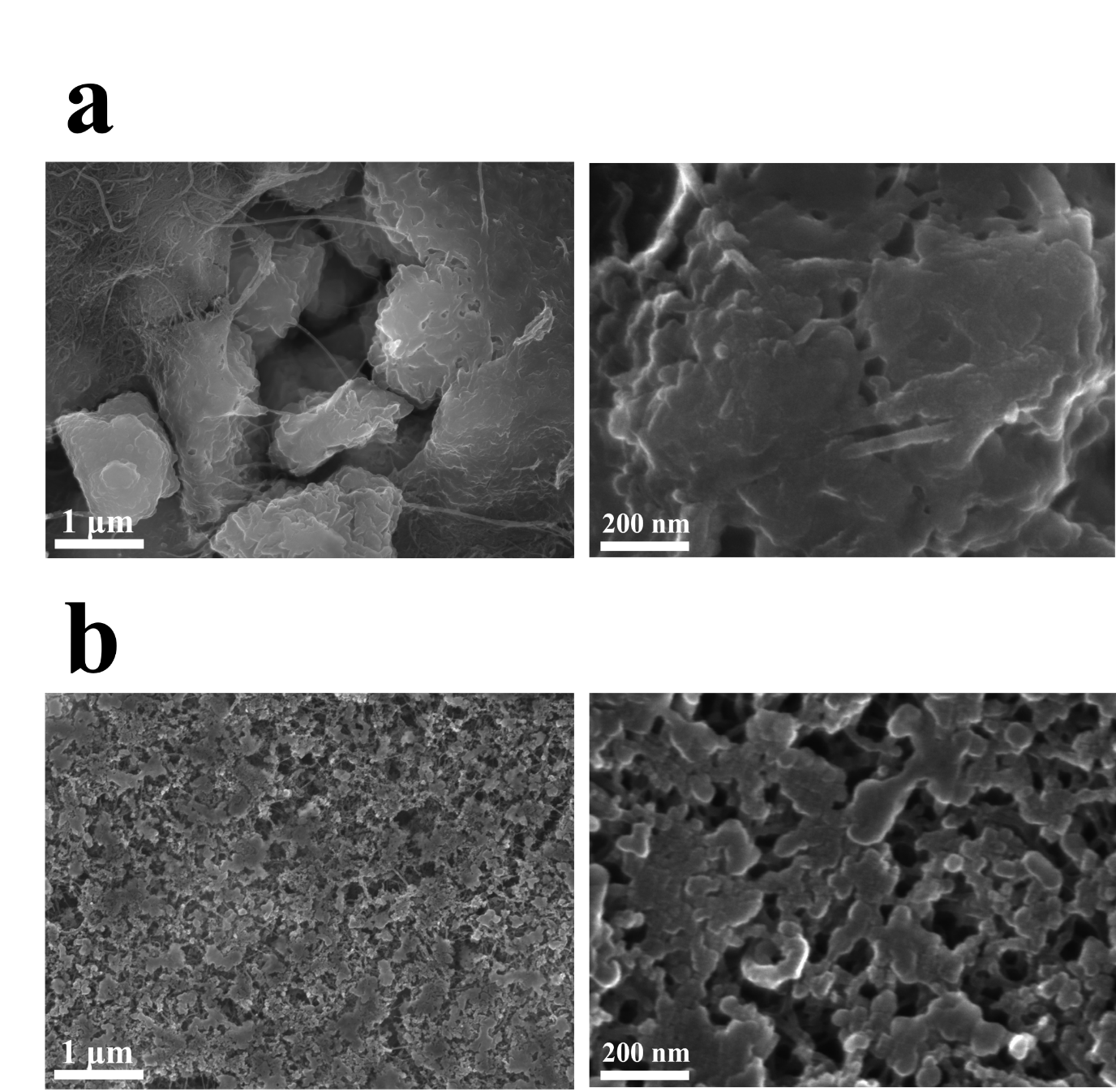


Figure S1. SEM images of the (a) PVDF and (b) PAN electrodes before electrochemical testing.


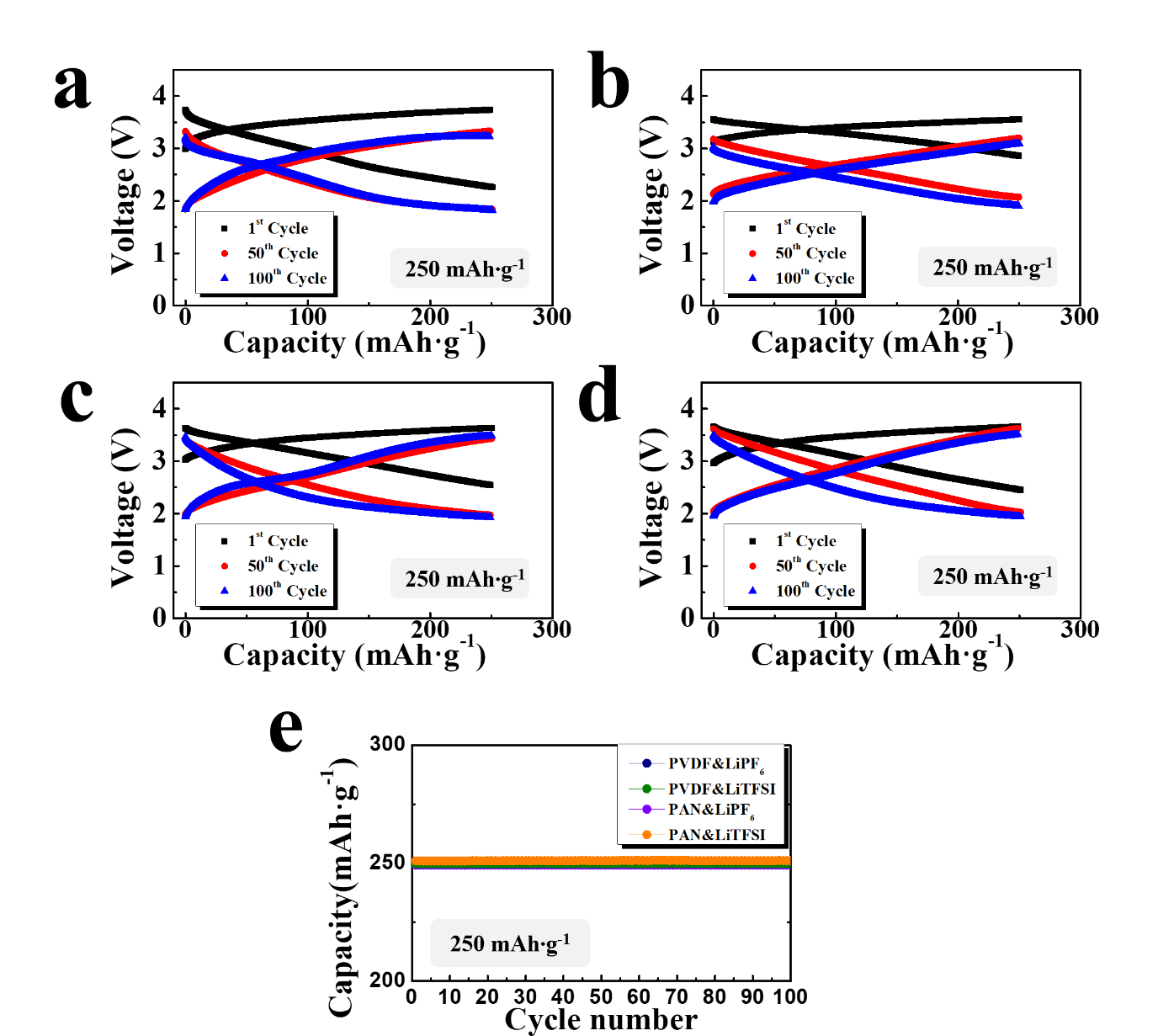


Figure S2. Charge–discharge profiles of the electrode measured with a limiting capacity of 250 mAh·g^−1^: (a, b) PVDF electrodes cycled using the (a) LiPF_6_ and (b) LiTFSI electrolytes; (c, d) PAN electrodes cycled using the (c) LiPF_6_ and (d) LiTFSI electrolytes; (e) cyclic performance of all four cells with the limiting capacity of 250 mAh·g^−1^.


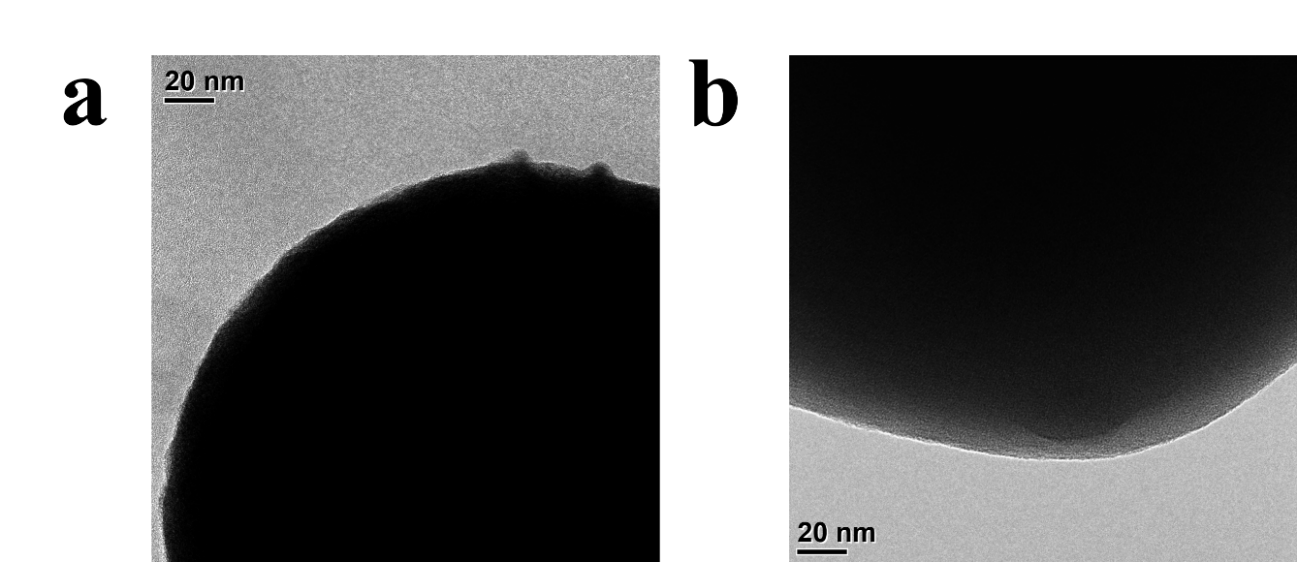


Figure S3. TEM images of the (a) PVDF and (b) PAN electrodes before electrochemical testing.


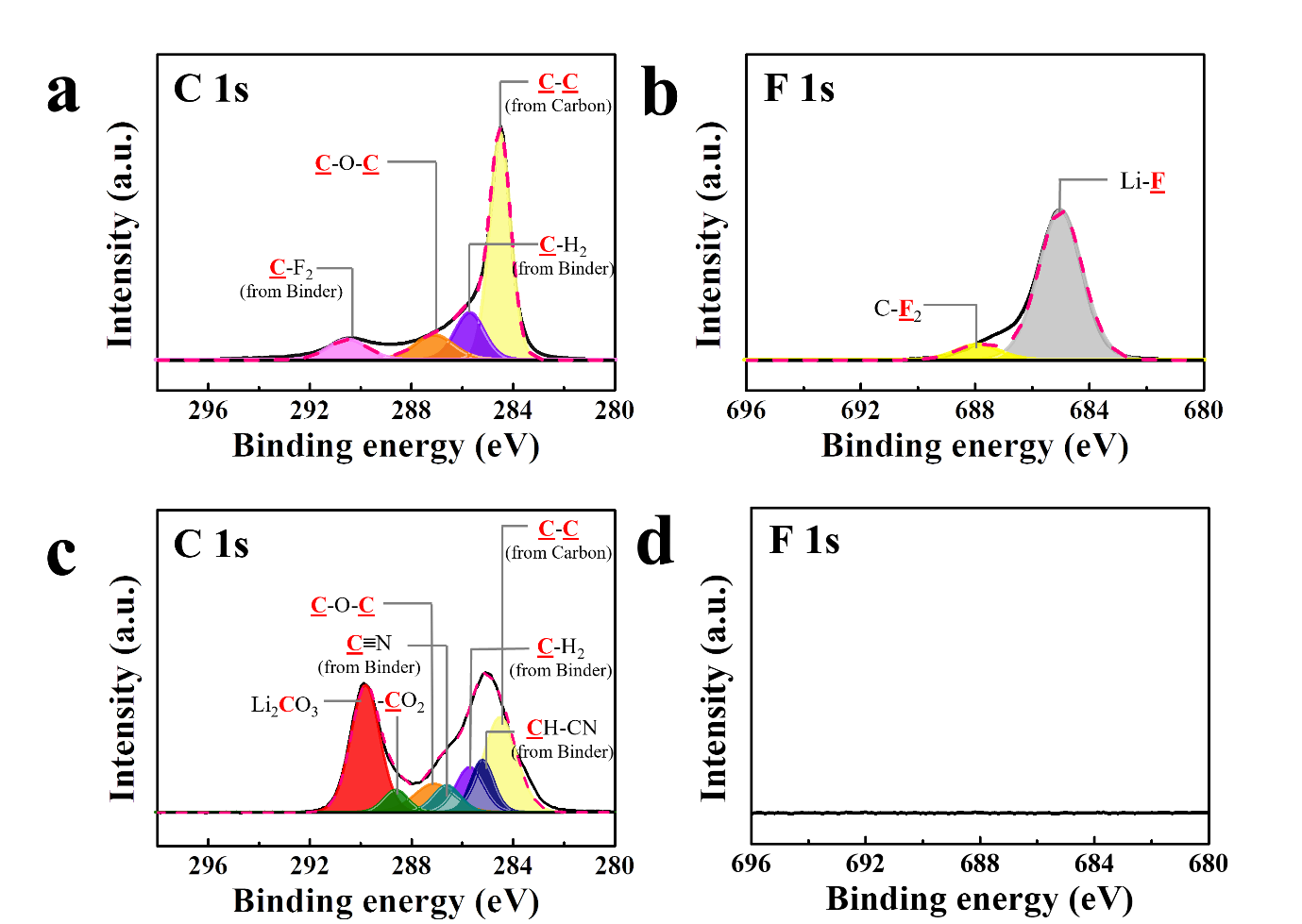


Figure S4. XPS spectra of pristine electrodes before electrochemical testing: (a) C 1s and (b) F 1s spectra of the PVDF electrode; (c) C 1s and (d) F 1s spectra of the PAN electrode.


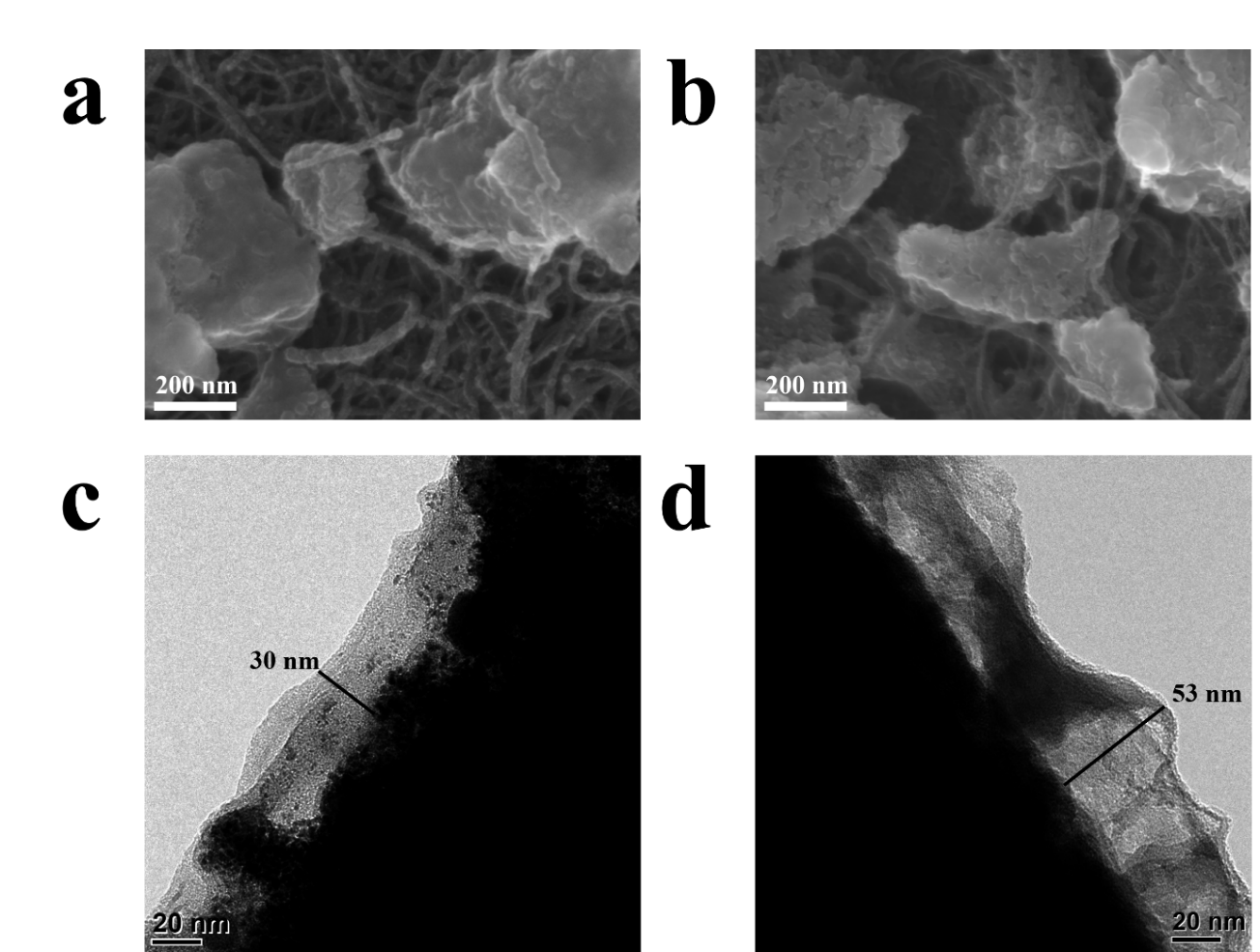


Figure S5. Surface morphology of the PVDF electrode after cycling with the LiPF_6_ electrolyte with a limiting capacity of 300 mAh·g^−1^; SEM images after (a) 1 and (b) 100 cycles; TEM images after (c) 1 and (d) 100 cycles.


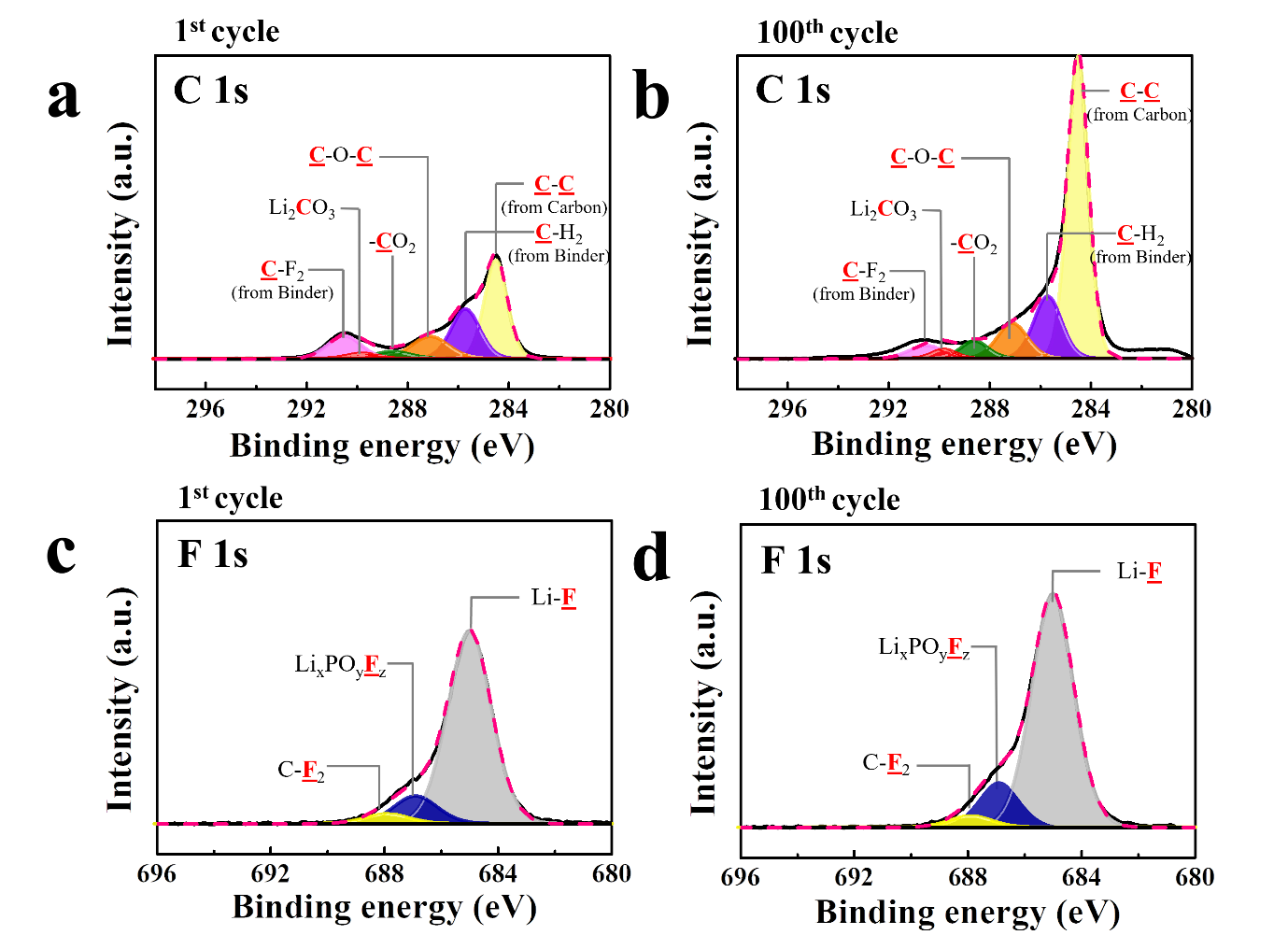


Figure S6. XPS spectra of PVDF electrode cycled using the LiPF_6_ electrolyte: C 1s spectra after the (a) 1^st^ and (b) 100^th^ cycles; F 1s spectra after the (c) 1^st^ and (d) 100^th^ cycles.


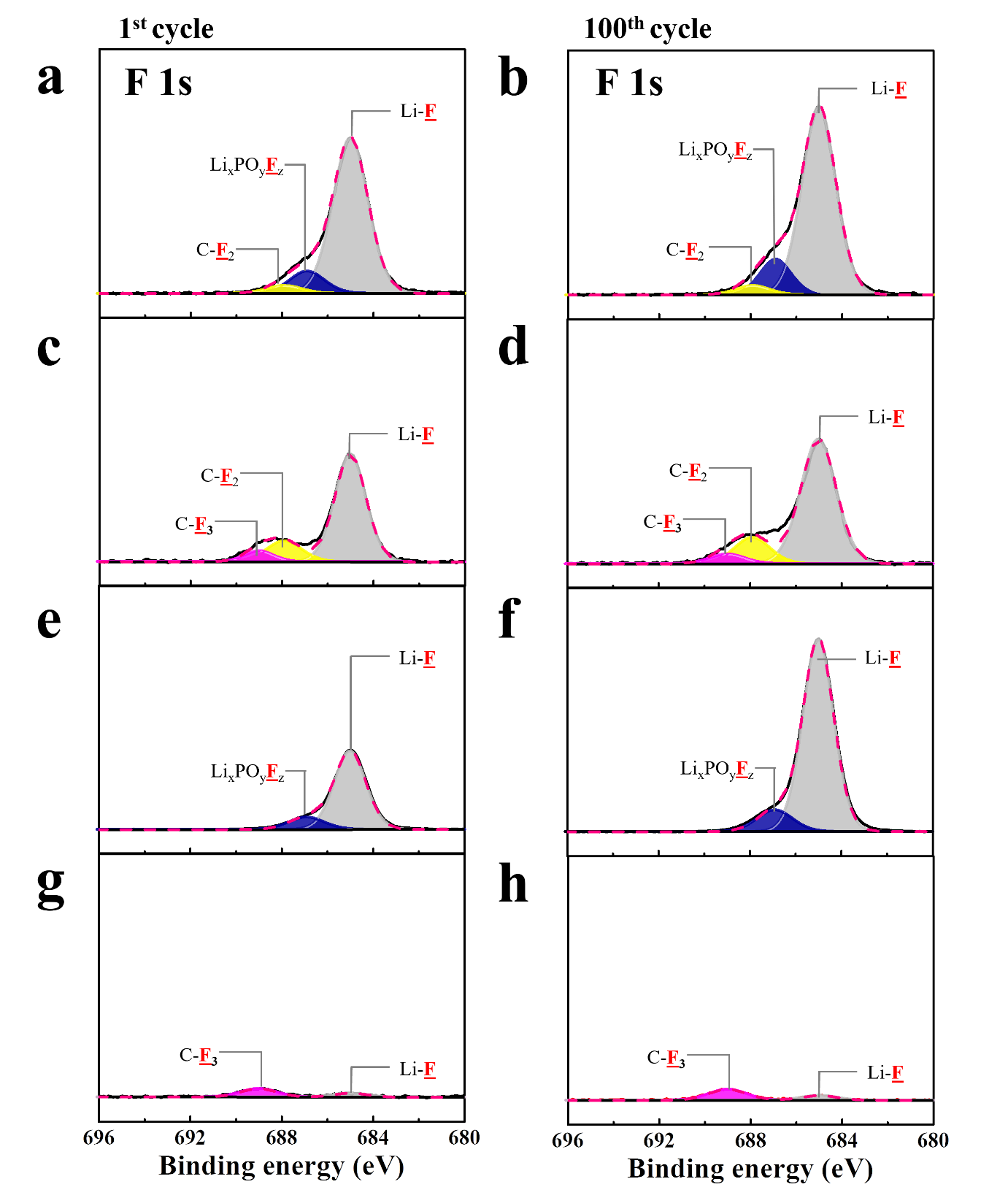


Figure S7. Comparison of the F 1s XPS spectra of the electrodes after 1 (left) and 100 cycles (right). PVDF electrode cycled using the (a, b) LiPF_6_ and (c, d) LiTFSI electrolytes; PAN electrode cycled using the (e, f) LiPF_6_ and (g, h) LiTFSI electrolytes.
